# Effectiveness of two supportive periodontal care protocols and outcome predictors during periodontitis: A randomized controlled trial

**DOI:** 10.1002/jper.70007

**Published:** 2025-11-07

**Authors:** Gaetano Isola, Marco Annunziata, Angela Angjelova, Angela Alibrandi, Gianluca Martino Tartaglia, Frank A. Scannapieco

**Affiliations:** ^1^ Department of General Surgery and Medical‐Surgical Specialties, Unit of Periodontology University of Catania Catania Italy; ^2^ International Research Center on Periodontal and Systemic Health “PerioHealth” University of Catania Catania Italy; ^3^ Multidisciplinary Department of Medical‐Surgical and Dental Specialties University of Campania “Luigi Vanvitelli” Naples Italy; ^4^ Department of Economics, Unit of Statistics University of Messina Messina Italy; ^5^ Fondazione IRCCS Cà Granda Ospedale Maggiore Policlinico Milan Italy; ^6^ Department of Biomedical, Surgical, and Dental Sciences University of Milan Milan Italy; ^7^ Department of Oral Biology, School of Dental Medicine University at Buffalo, The State University of New York Buffalo New York USA

**Keywords:** bleeding on probing, clinical trial, inflammation, periodontal treatment, periodontitis, smoking, supportive periodontal care

## Abstract

**Background:**

A randomized, controlled trial was conducted to evaluate the effectiveness of two supportive periodontal care (SPC) approaches in patients with periodontitis and to evaluate possible predictors influencing bleeding on probing (BoP) changes at 24‐month follow‐up.

**Methods:**

Fifty‐six periodontitis patients who first received active periodontal treatment by means of quadrant‐wise subgingival instrumentation were subsequently assigned to either a control group (oral hygiene instruction with supragingival instrumentation and dental polishing, *n* = 28) or a test group (oral hygiene instruction with both supra‐ and subgingival instrumentation and dental polishing, *n* = 28). BoP was the primary outcome, and probing pocket depth (PPD), clinical attachment level (CAL), full‐mouth plaque score (FMPS), gingival bleeding index (GBI), and the number of pocket sites were secondary outcome measures, recorded up to 24 months of follow‐up. A mixed generalized linear regression analysis also assessed the potential confounding factors that influenced BoP changes at 24 months.

**Results:**

At 24 months, both groups showed significant improvement in periodontal outcomes (*p* < 0.05). The test intervention was more effective than the control in reducing median BoP (*p* = 0.033), GBI (*p* = 0.023), the number of pockets ≥4 mm with BoP (*p* = 0.018), 4–5 mm (*p* = 0.048), 5–6 mm (*p* = 0.011), and >6 mm (*p* = 0.023). Among all follow‐up sessions, the reduced BoP was significantly negatively influenced by the number of median PPD (*p* = 0.031), the number of pockets 4–5 mm (*p* = 0.029), PPD 5–6 mm (*p* = 0.036), smoking (*p* = 0.039), and by the number of cigarettes/day (*p* = 0.042) and positively by test treatment (*p* = 0.033).

**Conclusion:**

SPC that included subgingival instrumentation yielded better results than the control to reduce BoP at 24‐month follow‐up. Smoking and deep pockets negatively influenced the BoP reduction in patients who underwent SPC.

**Plain Language Summary:**

Supportive periodontal care (SPC) is a series of individualized, site‐specific treatments aimed at preventing periodontitis recurrence/progression after successful completion of active periodontal therapy. SPC approaches performed by means of oral hygiene instruction with supra‐ and subgingival instrumentation and dental polishing (test group) compared to oral hygiene with supragingival instrumentation alone and dental polishing (control group) were both effective in reducing median periodontal outcomes, such as bleeding on probing (BoP), probing pocket depth (PPD), clinical attachment level (CAL), and full‐mouth plaque score (FMPS) after active periodontal treatment. However, the SPC approach that included subgingival instrumentation yielded better results compared to oral prophylaxis alone in reducing BoP, a parameter that was significantly influenced also by smoking, median PPD, a high number of sites with PPD 4–5 mm, 5–6 mm, and no sites >6 mm at 24‐month follow‐up.

## 1 INTRODUCTION

Periodontitis is a chronic, multifactorial inflammatory disease caused by tooth‐borne biofilm that, if not properly treated, results in an immune‐inflammatory response that could lead to the destruction of tooth‐supporting tissues and tooth loss.^1^ It has been widely demonstrated that periodontitis is negatively associated with several systemic conditions,^2^, ^3^ and that proper management of the disease starts with patient motivation and instruction in oral hygiene (Step 1 of periodontal treatment), accompanied by nonsurgical periodontal treatment (NSPT), the removal of the supra‐ and subgingival bacterial deposits (Step 2) from the teeth. This intervention often results in stable periodontal outcomes, including the reduction in bleeding on probing (BoP), probing pocket depth (PPD),^4^ and the number of deep periodontal pockets.^5^, ^6^, ^7^ In this regard, recently published guidelines of the European Federation of Periodontology (EFP) suggest that the treatment of periodontitis should be followed by a supportive periodontal care (SPC) program aimed at stabilizing the results of active periodontal treatment through tailored, site‐specific, individually designed interventions.^8^ SPC encompasses a bundle of preventive and therapeutic interventions rendered at various intervals tailored according to the patient´s risk profile and periodontal conditions. After active therapy is completed, site‐specific routine professional mechanical supra‐ and subgingival plaque removal limits the rate of periodontal attachment loss and enhances periodontal stability/improvement.^9^ Together with proper lifestyle choices such as refraining from smoking, SPC has been reported to effectively prevent periodontitis progression over the long term.^10^

Studies comparing different SPC protocols have reported conflicting results. A review analyzing the effectiveness of SPC approaches showed that a SPC regimen of supragingival tooth cleaning alone or combined with subgingival instrumentation yielded comparable PPD at 1‐year follow‐up.^11^ Another recent randomized clinical trial in periodontitis patients concluded that SPC with or without subgingival instrumentation results in comparable subgingival microbiological outcomes up to 2 years of follow‐up.^12^, ^13^ However, a pioneering study^14^ reported excellent long‐term clinical outcomes and reduced tooth loss persisting up to 30 years of follow‐up in those who, after completion of periodontal therapy, maintained good gingival health status (determined as bleeding on probing [BoP] in <10% of sites and treatment of sites PPD ≥4 mm BoP positive) and treated residual pockets when necessary with subgingival instrumentation.^5^ In this regard, among different periodontal parameters, it has been reported that BoP could be a valuable indicator of periodontitis activity monitoring and progression risk, as it has been reported as one of the cardinal symptoms reflecting periodontal tissue inflammation.^15^ In this regard, a recent randomized clinical trial^16^ reported that BoP was reported as a worthy predictor variable for the evaluation of SPC treatments in periodontitis patients at 1‐year follow‐up.

Smoking is considered^17^, ^18^ one of the most important habits that increases the risk for progression of periodontitis, causing decreased vascular flow, reduced immunoglobulin and lymphocyte production, and increased prevalence of periodontal pathogens.^19^ Through its ability to suppress inflammation, smoking also sometimes masks early clinical signs such as BoP and mucosal redness,^20^ thus complicating the diagnosis and effectiveness of periodontal treatment in the short‐term^21^ and, most importantly, the efficacy of SPC programs in the long‐term by reducing periodontal vascularity and the overall host response.^22^

The present study compared the clinical efficacy of two SPC protocols in patients with periodontitis at 24‐month follow‐up after active treatment, using BoP as the primary outcome variable. Furthermore, the effect of other predictors on the efficacy of the two SPC protocols at 24 months after treatment was evaluated.

## 2 MATERIALS AND METHODS

### 2.1 Study design

The study was designed as a two‐arm, non‐inferiority, randomized controlled clinical trial to compare two SPC approaches with a 2‐year follow‐up. Patients with periodontitis diagnosed according to the established case definitions^1^ and candidates for periodontal therapy were first treated through conventional quadrant‐wise NSPT. Starting 3 months from the last active periodontal treatment session, participants were assigned to one of two SPC groups: (1) the test group, which included oral hygiene instruction with both supra‐ and subgingival instrumentation and dental polishing, or (2) the control group, that included oral hygiene instruction with only supragingival instrumentation and dental polishing, The study was conducted in observance of the Principles of the Declaration of Helsinki on experimentation involving human subjects. Written consent was obtained from all patients, and the research protocol was reviewed and approved by the Institutional Review Board of the University of Catania (approval numbers: 215/PO and 24/20/PAR). The study followed the CONSORT guidelines (Table S1 in online *Journal of Periodontology*), and the study protocol was registered on clinicaltrials.gov (NCT06382753). The null hypotheses were (1) no statistical differences between SPC approaches in terms of BoP reduction, and (2) no predictors that influenced the efficacy of SPC approaches were tested.

### 2.2 Sample size calculation

The power calculation was determined using statistical software.* The sample size was calculated using BoP as a primary outcome^15^, ^23^ and considering two groups, a power level of 80%, an effect size of 0.80, and a 2‐sided level of 5%. It was fixed a priori that at least 26 subjects per group were needed to achieve a power level of 80%. However, 28 subjects per group were targeted for enrolment to avoid potential dropouts during follow‐up sessions.

### 2.3 Study participants

All subjects were recruited from the Unit of Periodontology, School of Dentistry, at the University of Catania, Catania, Italy. The inclusion criteria were: (1) age ≥18 years old; (2) having ≥16 natural teeth; (3) patients with a diagnosis of periodontitis^1^; (4) PPD of ≥4 mm and clinical attachment level (CAL) of ≥2 mm in at least 40% of the periodontal sites; (5) a full‐mouth bleeding score (FMBS) of at least 40%; (6) at least two sites with bone loss of ≥2 mm as assessed radiographically by periapical x‐rays.

The exclusion criteria were: (1) use of contraceptive drugs 6 months or less before the study; (2) use of anti‐inflammatory drugs, immunosuppressants, or antibiotics 6 months or less before the study; (3) pregnancy; (4) history of heavy alcohol intake (eight or more drinks per week for a woman or 15 or more drinks per week for a man); (5) allergy or intolerance to drugs; (6) any periodontal treatment 6 months before the study; (7) any systemic disease which could impact the study results.

### 2.4 Outcome measures

All periodontal parameters were assessed at six sites (mesio‐buccal, buccal, disto‐buccal, disto‐oral, oral, and mesio‐oral) using a manual periodontal probe† with a probing force of nearly 0.2 N by a calibrated examiner (A.A.). The primary outcome evaluated was the BoP reduction, measured dichotomously as positive/negative if bleeding occurred within 30 seconds after probing using a manual periodontal probe.^‡,^ ^23^ Secondary outcomes included the evaluation of the gingival bleeding index (GBI) based on the gingival index,^24^ PPD, the number of teeth present, and full‐mouth plaque score (FMPS) stained with a disclosing agent (recorded as present or absent).^25^ CAL was calculated using the CEJ as a reference. Moreover, the percentage of sites with a PPD ≥4 mm that were BoP positive was also recorded.

How smoking and the number of cigarettes/day influenced the efficacy of SPC protocols was also evaluated. Patients who reported smoking were classified as current smokers, ex‐smokers (cessation ≥5 years), or never smokers.^26^ Moreover, for SPC compliance, patients were categorized as regular (present at every follow‐up session and adhering to all prescribed treatments), erratic (present at every follow‐up session and not fully adhering to all prescribed treatments) and non‐compliant (present at every follow‐up and not adhering to any prescribed treatment) based on single adherence to scheduled appointments.^27^

### 2.5 Randomization, calibration, and masking

For the study, patients were randomly allocated to the SPC groups using sealed and numbered envelopes. Details of the randomization sequence for patient group allocation were concealed from a clinician (A.A.) who was blinded and had not participated in the subsequent study steps. A random allocation sequence in a 1:1 ratio using a computer generator where delivered in sealed envelopes to clinicians who performed the instruction, motivation, and treatment procedures.

One examiner (M.A.) attended a training and calibration session with a total of six patients not involved in the trial using a periodontal probe.^*^ Both intra‐ and inter‐examiner reliability were assessed using BoP as the outcome variable. Probing consistency was considered sufficient if the percentage of agreement within ±5% for BoP between repeated measurements was at least 95%; in this case, the agreement was 93.8%. For the intra‐examiner reliability, the kappa coefficients were calculated, reporting high reliability for both examiners, the first (*k* = 0.81) and the second (*k* = 0.83). The intraclass correlation coefficient (ICC = 0.822) was calculated for the inter‐examiner reliability. The calibrated examiners were unaware with respect to the test or control procedure.

### 2.6 Active treatment stage

All patients received an initial supragingival instrumentation session. They also received individualized oral hygiene instructions, which included interdental plaque control with interproximal brushes (tailored to each patient) and toothbrushing using a modified Bass technique.^13^ All of the participants were provided with the same type of toothpaste‡, toothbrush, and interdental brushes.§ ^,^ ‖

NSPT was performed using Gracey curettes (1/2, 5/6, 7/8, 11/12, and 13/14) and an ultrasonic device with inserts (No. #1, 2#, and #1S) according to the operator preference.¶ The ultrasonic device was used with constant water irrigation and a frequency of 20 kHz at a power setting of 60 watts. All patients received traditional NSPT through a quadrant‐wise approach in four different sessions with an interval of 1 week between each session. For each patient, the first session was initiated in the upper right maxillary quadrant. No antibiotics or other medications were prescribed after treatment. At the end of each treatment session, patients were reinstructed and motivated to perform personal oral hygiene with both toothbrushes and interdental toothbrushes.^‖^ The last active periodontal treatment session occurred 6 months after baseline.

### 2.7 Supportive periodontal treatment stage

After 3 months from the last active periodontal treatment session (representing the SPC baseline), the subjects assigned to the control group received oral hygiene instructions, full‐mouth supragingival debridement, and dental polishing. The subjects in the test group, the same treatment as the control group was provided, plus a full‐mouth subgingival debridement aimed at gently removing visible or detectable subgingival deposits located on the crown of the tooth, performed using both hand and ultrasonic instruments (tips No. #1, 2#, and #1S^#^ used with constant water irrigation with a 20.000 Hz frequency).

In both groups, at each SPC follow‐up session, oral hygiene measures were reinforced through hygiene instructions, which included interdental plaque control with interproximal brushes^‖^ (tailored to each patient) and toothbrushing using a modified Bass technique. No mouthwashes were prescribed.

Patients were excluded from the study if they experienced worsening of disease progression identified as a change of CAL ≥3 mm in at least two sites, which was set as the threshold for disease progression.^28^ These excluded subjects were re‐treated. The clinical examination was performed at SPC baseline and follow‐up intervals of 3, 6, 12, 18, and 24 months from SPT baseline.

### 2.8 Statistical analysis

Quantitative outcomes were expressed as median and interquartile range (Q1–Q3), and categorical variables as absolute frequencies and percentages. A nonparametric approach was applied because the distribution of quantitative outcomes was not Gaussian, as verified by the Shapiro–Wilk test. In order to perform the inter‐group comparison, the Mann–Whitney test was applied for quantitative outcomes and the chi‐squared test for categorical variables. For each quantitative outcome, a comparison among time points (Baseline, 3, 6, 12, 18, and 24 months after NSPT) was performed by using the Friedman test for both groups separately (intragroup analysis), followed by a 2‐by‐2 comparison performed using the Tukey post‐hoc test. To control the increased risk of Type I error due to multiple comparisons, the Bonferroni correction was applied, whereby the significant alpha level of 0.050 was divided by the number of possible comparisons for the active phase of treatment (4 follow‐up sessions, 6 possible comparisons) and the subsequent SPC phase of treatment (6 follow‐up sessions, 15 possible comparisons). Thus, the “adjusted” significance level for this analysis was 0.050/6 = 0.008 for the active phase of treatment and 0.050/15 = 0.003 for the SPT phase of treatment.

For the analyses, the patient was set as the test unit. The primary outcome was the BoP reduction, while the secondary outcome assessed the reduction of PPD, CAL, FMPS, GBI, FMBS, number of teeth, number of sites with PPD <4 mm, PPD 4–5 mm, PPD 5–6 mm, PPD > 6 mm, % sites PPD <4 mm, % sites PPD 4–5 mm, % sites PPD 5–6 mm and % sites PPD > 6 mm and the number of sites with a PPD ≥4  mm which were BoP positive, both at 24‐month follow‐up during SPC. As an exploratory analysis, the influence of possible clinical predictors of the BoP changes was evaluated at all follow‐up sessions.

Finally, a mixed generalized linear regression model was estimated in order to evaluate significant predictors of BoP changes at all follow‐up sessions (SPT baseline, 3, 6, 12, 18, and 24 months). The confounding effects were represented by the following covariates such as treatment (control group treatment as a reference), time of treatment (baseline as a reference), age, sex (male/female), smoking, number of cigarettes/day, gingival biotype (thin/thick), patient compliance (regular/erratic or not‐compliant), PPD, CAL, FMPS, number of sites with PPD <4 mm, PPD 4‐5 mm, PPD 5–6 mm, PPD > 6 mm, % sites PPD <4 mm, % sites PPD 4–5 mm, % sites PPD 5–6 mm and % sites PPD > 6 mm. Sex and gingival biotypes were considered at baseline, while other parameters were considered at all follow‐up sessions. Firstly, uni‐level models were estimated, in which a single predictor was tested against the primary outcome (BoP at all follow‐up sessions). Then, a multi‐level model was estimated by inserting only the variables that were found to be statistically significant in the uni‐level models. Specifically, the potential predictor variables were tested together. Moreover, the Variance Inflation Factor (VIF) index was calculated to verify the low degree of multicollinearity existing between the various confounding variables. Both uni‐ and multi‐level model results were reported as estimation coefficients, 95% confidence intervals (CI), and *p*‐values.

Statistical analyses were performed using statistical software.# A *p*‐value < 0.05 was considered to be statistically significant.

## 3 RESULTS

### 3.1 Sample characteristics

**FIGURE 1:**
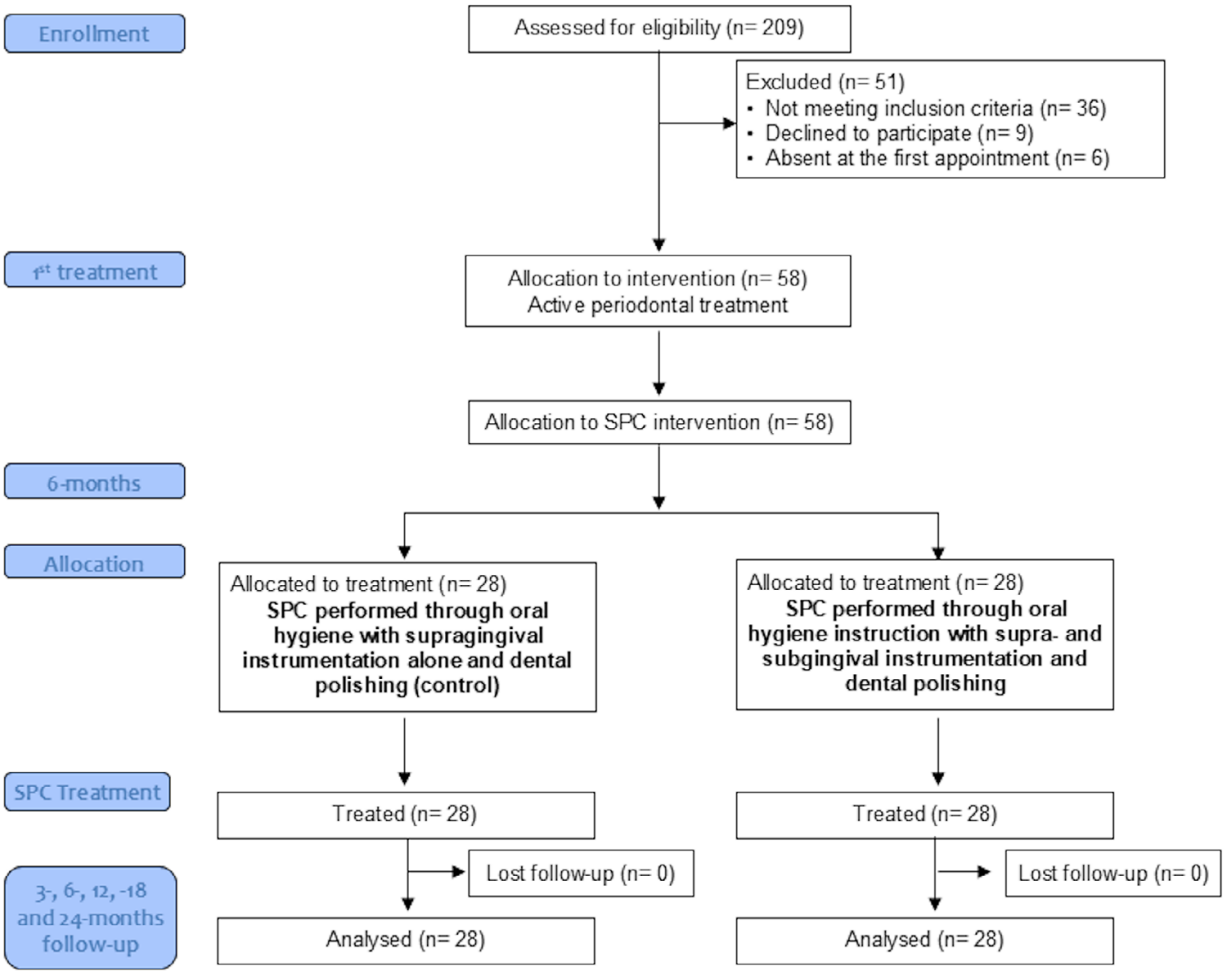
Study flowchart

A total of 109 periodontitis patients were screened, after which 53 subjects were excluded because they did not meet the inclusion criteria (*n* = 38), refused participation in the study (*n* = 9), or were absent for the first visit (*n* = 6) (Figure 1). A final number of 56 subjects (28 per group) were included for analysis and classified for periodontitis staging and grades. Patients were well balanced for age (*p* = 0.347), sex (*p* = 0.479), and periodontitis staging and grading (*p* > 0.05) (Table 1). None of the patients were lost during the follow‐up sessions, and no complications were noted.

**TABLE 1:** Clinical characteristics of the analyzed sample.

Parameter	Control group (*n* = 28)	Test group (*n* = 28)	*p*‐value
Sex (male), no. (%)	15 (53.6)	16 (57.1)	0.336
Age, median (IQR)	51.6 (47.5–56.9)	52.1 (48.3–54.9)	0.478
**Race, no. (%)**			
Caucasian	28 (100)	28 (100)	0.941
**Patient's education level, no. (%)**			0.441
Primary School	13 (46.4)	12 (42.9)	
High School	10 (35.7)	11 (39.3)	
University	5 (17.9)	5 (17.9)	
BMI (kg/m^2^), median (IQR)	21.8 (18.6–22.9)	20.9 (17.9–23.1)	0.314
**Patient's compliance, no. (%)**			
Regular	15 (53.6)	14 (50)	0.331
Erratic	9 (32.1)	10 (35.7)	0.258
Non‐compliers	4 (14.3)	4 (14.3)	0.951
**Smoking, no. (%)**			
Never smokers	14 (50)	13 (46.4)	0.745
Ex‐smokers	4 (14.3)	6 (21.4)	0.155
Current smokers	10 (35.7)	9 (32.1)	0.556
No. cigarettes/day, median (IQR)	11.1 (8.5–12.9)	10.6 (8.1–13.5)	0.657
**Stage and grade periodontitis, no. (%)**			
Stage III periodontitis	17 (60.7)	16 (57.1)	0.287
Grade A	10 (35.7)	9 (32.1)	0.698
Grade B	4 (14.3)	5 (17.9)	0.856
Grade C	3 (10.7)	3 (10.4)	0.954
Stage IV periodontitis	11 (39.3)	12 (42.9)	0.331
Grade A	6 (21.4)	6 (21.4)	0.921
Grade B	4 (14.3)	3 (10.7)	0.745
Grade C	1 (3.6)	2 (7.1)	0.647
**Extent of periodontitis, no. (%)**			
Localized (<30% of teeth involved)	10 (35.7)	12 (42.9)	0.214
Generalized (>30% of teeth involved)	18 (64.3)	16 (57.1)	0.589
Number of teeth, median (IQR)	22.3 (20.4–25.6)	22.8 (19.5–25.9)	0.206
KT, mm, median (IQR)	2.56 (2.16–2.89)	2.44 (2.05–2.79)	0.339
**Gingival biotype, no. (%)**			
Thin gingival biotype	16 (57.1)	15 (53.6)	0.417
Thick gingival biotype	12 (42.9)	13 (46.4)	0.319

*Note*: Results are represented as median and interquartile range (IQR) or number and percentage.

Abbreviations: BMI, body mass index; IQR, interquartile range; KT, keratinized tissue.

### 3.2 Active phase of periodontal treatment

Compared to the baseline, active periodontal treatment significantly reduced the median PPD, CAL, BoP, and FMPS (Table S2 in online *Journal of Periodontology*). At 6‐months, patients had a reduction in the median PPD (3.35 mm, *p* = 0.006), as well as in the number of pockets with PPD ≥4 mm (*p* = 0.027), PPD ≥5 mm (*p* = 0.044), PPD ≥6 mm (*p* < 0.001), in the number of sites with PPD ≥4 mm BoP+ (*p* = 0.025), in the % PPD < 4 mm (*p* = 0.048), % PD ≥4 mm (*p* = 0.029), mean CAL (*p* = 0.021), FMBS (*p* = 0.041), and FMPS (*p* = 0.017) (Table S2).

### 3.3 Results of the SPC: Primary outcome

At SPC baseline, there were no differences among groups for all analyzed parameters (Table 2). At the 24‐month follow‐up from the SPC baseline, both groups reported a significant reduction in median BoP, PPD, CAL, and FMPS (*p* < 0.05). At the same time, the other parameters, such as the number of teeth, were not significant (Table 2).

**TABLE 2:** Clinical values of periodontal parameters in both groups at baseline and at each follow‐up session at the end of the active stage of treatment.

	Control group (*n* = 28)	Test group (*n* = 28)	
Timepoints	Median (IQR)	Median (IQR)	*p*‐value intergroup
**BoP (%)**			
Baseline	32.5 (28.9–34.6)	33.6 (27.4–36.5)	0.335
3 months	26.4 (24.4–29.6)^*^	24.3 (17.4–26.5)^*^	0.066
6 months	28.4 (25.6–32.3)	20.5 (17.5–23.4)	0.036
12 months	25.6 (22.2–27.6)	21.4 (18.5–23.1)	0.019
18 months	24.6 (22.1–27.9)	20.1 (16.5–21.9)	0.041
24 months	24.5 (22.1–26.5)	19.2 (18.6–24.5)	0.033
Δ 0–24 months	8 (6.5–9.5)	14.4 (11.6–17.7)	0.006
Intragroup *p*‐value	0.018	0.026	
**PPD (mm)**			
Baseline	3.42 (2.8–3.9)	3.34 (2.7–3.7)	0.341
3 months	2.99 (2.7.3.2)	2.75 (2.6–3.2)^*^	0.042
6 months	2.98 (2.7–3.6)	2.85 (2.6–3.3)	0.139
12 months	3.19 (2.9–3.8)	3.03 (2.8.3.4)	0.226
18 months	3.29 (2.9–3.3)	2.81 (2.6–3.1)^*^	0.026
24 months	3.12 (2.8–3.4)	2.71 (2.5–3.1)^*^	0.009
Δ 0–24 months	0.3 (0.1–0.4)	0.6 (0.3–0.8)	0.039
Intragroup *p*‐value	0.022	0.034	
**CAL (mm)**			
Baseline	3.59 (3.1–3.7)	3.53 (3.1–3.7)	0.554
3 months	3.19 (3–3.5)	2.82 (2.5–3.3)^*^	0.048
6 months	3.11 (2.9–3.3)	3.12 (2.9–3.4)	0.087
12 months	3.35 (3.3–3.7)	2.96 (2.7–3.4)	0.025
18 months	3.31 (3.1–3.9)	2.85 (2.6–3.5)^*^	0.031
24 months	3.22 (2.9–3.5)	2.79 (2.5–3.1)^*^	0.017
Δ 0–24 months	0.3 (0.2–0.5)	0.5 (0.3–0.6)	0.042
Intragroup *p*‐value	0.032	0.022	
**FMPS (%)**			
Baseline	18.3 (15.4–21.3)	18.9 (15.5–21.5)	0.255
3 months	15.9 (13.2–16.8)^*^	14.4 (11.6–18.5)^*^	0.336
6 months	16.6 (15.2–21.5)	13.2 (12.1–19.6)	0.024
12 months	17.5 (14.3–22.3)	13.9 (12.3–18.6)	0.028
18 months	19.5 (16.5–21.5)	13.5 (11.5–18.4)	0.033
24 months	17.4 (16.9–21.1)	14.2 (12.7–17.8)^*^	0.029
Δ 0–24 months	0.9 (0.6–1.5)	4.7 (3.2–6.6)	0.002
Intragroup *p*‐value	0.014	0.039	
**GBI (%)**			
Baseline	11.6 (8.9–13.5)	12.1 (8.7–14.3)	0.471
3 months	9.5 (7.2–11.5)	8.4 (7.9–11.5)^*^	0.109
6 months	12.9 (10.5–15.1)	7.6 (7.1–11.5)	0.014
12 months	12.1 (9.2–14.6)	8.5 (7.4–11.9)	0.047
18 months	11.9 (9.2–13.9)	8.9 (7.8–10.5)	0.055
24 months	10.5 (8.2–14.3)	8.1 (7.8–13.6)	0.023
Δ 0–24 months	1.1 (0.9–1.8)	4 (3.1–4.9)	0.017
Intragroup *p*‐value	0.01	0.019	
**Number of teeth**			
Baseline	22.3 (19.6–25.4)	21.9 (19.5–24.9)	0.336
3 months	22.2 (20.6–23.5)	22.7 (21.9–23.6)	0.295
6 months	22.1 (19.5–22.9)	22.5 (20.5–23.5)	0.445
12 months	21.9 (19.1–24.3)	22.1 (20.9–23.9)	0.578
18 months	21.8 (19–24.4)	21.6 (20.5–23.5)	0.749
24 months	21.7 (18.9–23.3)	21.5 (20.1–23.2)	0.567
Δ 0–24 months	0.6 (0.4–0.9)	0.4 (0.2–0.7)	0.347
Intragroup *p*‐value	0.226	0.657	
**No. pockets PPD < 4 mm**			
Baseline	21.2 (18.5–23.4)	20.5 (18.5–22.5)	0.652
3 months	15.6 (11.9–17.9)^*^	13.1 (9.5–14.5)^*^	0.241
6 months	17.5 (14.5–18.5)	14.1 (13.2–15.5)	0.105
12 months	16.1 (14.6–19.5)	13.9 (12.4–16.2)	0.066
18 months	18.5 (15.6–21.2)	16.1 (12.3–18.5)^*^	0.041
24 months	19.9 (17.5–21.5)	17.5 (11.9–19.5)	0.049
Δ 0–24 months	2.3 (1.7–2.9)	3 (2.5–6.4)	<0.001
Intragroup *p*‐value	0.105	0.032	
**No. pockets PPD ≥ 4 mm BoP+**			
Baseline	14.3 (11.2–18.5)	15.1 (13.2–16.5)	0.332
3 months	7.5 (6.5–8.9)^*^	7.1 (5.5–9.5)^*^	0.741
6 months	9.6 (7.4–11.5)	8.2 (7.6–10.4)^*^	0.154
12 months	12.4 (9.5–14.2)	8.1 (7.1–10.9)^*^	0.059
18 months	13.6 (8.5–14.9)	9.1 (8.1–13.2)^*^	0.047
24 months	12.5 (9.6–13.8)	8.7 (7.4–9.6)^*^	0.018
Δ 0–24 months	1.8 (0.9–2.5)	6.4 (5.1–8.2)	0.005
Intragroup *p*‐value	0.021	0.048	
**No. pockets PPD 4–5** **mm**			
Baseline	19.6 (13.6–24.5)	18.8 (14.2–24.1)	0.324
3 months	18.9 (12.4–25.3)	11.7 (9.2–19.5)^*^	0.089
6 months	15.7 (9.1–18.4)	13.1 (9.5–18.3)	0.065
12 months	15.8 (11.6–23.4)	11.9 (8.7–15.2)^*^	0.014
18 months	17.2 (12.5–25.4)	9.7 (7.8–14.5)^*^	0.017
24 months	14.9 (10.5–18.4)^*^	11.2 (8.5–13.9)^*^	0.048
Δ 0–24 months	4.7 (2.4–7.5)	7.6 (6.1–11.5)	0.009
Intragroup *p*‐value	0.031	0.014	
**No. pockets PD 5–6** **mm**			
Baseline	15.2 (10.2–18.6)	15.9 (9.2–18.5)	0.475
3 months	13.8 (9.3–16.4)	11.2 (8.1–11.3)^*^	0.088
6 months	15.5 (11.3–17.8)	12.9 (9.1–14.6)	0.101
12 months	12.1 (9.1–16.3)	7.1 (5.1–8.7)^*^	0.037
18 months	13.7 (9.9–17.4)	7.4 (6–9.3)^*^	0.015
24 months	12.7 (9.1–15.9)	8.2 (6.1–9.3)^*^	0.011
Δ 0–24 months	2.5 (2.1–4.3)	7.7 (5.3–9.6)	0.041
Intragroup *p*‐value	0.033	0.011	
**No. pockets PPD > 6** **mm**			
Baseline	5.7 (2.9–6.2)	6.2 (4.5–7.2)	0.367
3 months	5.2 (2.7–6.1)	4.3 (3.5–5.7)	0.271
6 months	5.3 (3.3–7.5)	4.2 (3.2–5.9)	0.101
12 months	5.5 (4.2–6.2)	3.3 (2.9–4.1)^*^	0.044
18 months	5.7 (4.2–7.3)	3.2 (2.7–4.5)^*^	0.046
24 months	5.6 (3.9–6.4)	4.1 (2.7–3.9)	0.023
Δ 0–24 months	0.1 (0–0.2)	2.1 (1.5–2.9)	0.005
Intragroup *p*‐value	0.845	0.047	
**% PPD < 4** **mm**			
Baseline	77.5 (67.4–84.5)	78.5 (67.2–86.3)	0.381
3 months	81.2 (71.4–90.4)	85.3 (78.4–91.2)^*^	0.671
6 months	79.5 (67.8–93.5)	86.4 (76.6–95.3)	0.202
12 months	76.4 (66.8–84.3)	84.5 (71.3–91.4)	0.064
18 months	74.3 (66.7–81.2)	85.4 (78.3–89.4)	0.035
24 months	71.4 (61.2–78.9)^*^	83.3 (72.2–89.5)^*^	0.019
Δ 0–24 months	6.2 (5.1–7.4)	5.4 (2.3–7.5)	0.106
Intragroup *p*‐value	0.056	0.042	
**% PPD 4–5** **mm**			
Baseline	22.5 (17.6–26.7)	20.9 (16.5–25.6)	0.107
3 months	19.6 (16.5–24.4)	15.6 (10.2–18.9)^*^	0.088
6 months	18.9 (15.6–23.9)	16.1 (11.3–21.1)	0.205
12 months	18.4 (12.5–26.7)	15.5 (11.4–19.6)	0.057
18 months	18.1 (14.5.24.5)	13.2 (8.4–17.5)^*^	0.021
24 months	16.7 (13.3–21.4)^*^	11.4 (7.6–15.6)^*^	<0.001
Δ 0–24 months	5.8 (3.6–8.2)	9.3 (6.7–13.2)	0.004
Intragroup *p*‐value	0.034	0.002	
**% PPD 5–6** **mm**			
Baseline	7.2 (6.5–11.6)	7.9 (6.4–11.3)	0.285
3 months	6.7 (6.1–13.1)	5.1 (5.3–8.6)^*^	0.061
6 months	7.1 (5.1–11.3)	6.3 (5.1–7.3)	0.665
12 months	5.9 (4.1–7.3)	5.9 (4.5–7.8)	0.845
18 months	6.8 (5.7–8.3)	5.3 (5.5–7.6)	0.057
24 months	5.4 (4.1–8.6)	4.1 (3.2–5.1)^*^	0.048
Δ 0–24 months	1.8 (1.1–2.8)	3.3 (2.7–4.2)	0.025
Intragroup *p*‐value	0.018	0.035	
**% PPD > 6** **mm**			
Baseline	3.5 (2.6–4.4)	3.7 (2.6–4.3)	0.875
3 months	2.9 (2.1–3.3)	3.1 (2.1–4.4)	0.022
6 months	3.1 (2.2–3.6)	2.6 (1.8–3.7)	0.066
12 months	3.6 (2.4–4.8)	2.5 (1.8–3.4)	0.074
18 months	3.4 (2.7–4.2)	2.8 (2–3–6)	0.005
24 months	3.5 (2.5–4.7)	3.1 (2.1–4.4)	0.286
Δ 0–24 months	0.2 (0–0.4)	0.6 (0.3–0.9)	0.078
Intragroup *p*‐value	0.104	0.304	

*Note*: Results are presented as median (CI 95%). Bonferroni corrections between time points; ^*^significance between follow‐up sessions with a significant *p*‐value at adjusted alpha level of 0.003. *p* <0.05 among all follow‐up sessions.

Abbreviations: BoP, bleeding on probing; CAL, clinical attachment loss; GBI, gingival bleeding index; FMPS, full‐mouth plaque score; PPD, probing pocket depth.

Specifically, at 24 months, the test group showed better results than the control group with respect to median BoP (test, 19.2%; control, 24.5%, *p* = 0.033) and GBI (test, 8.1%; control, 10.5%, *p* = 0.023). Regarding PPD, the test group yielded a greater reduction of the number (no.) of pockets < 4 mm (test, 18.5; control, 17.5, *p* = 0.049), no. of pockets ≥4 mm BoP positive (test, 8.7; control, 12.5, *p* = 0.018), no. of pockets 4–5 mm (test, 11.2; control, 14.9, *p* = 0.048), no. of pockets 5–6 mm (test, 8.2; control, 12.7, *p* = 0.041), no. of pockets PPD > 6 mm (test, 4.1; control, 5.6, *p* = 0.023), and in the % PD 5‐6 mm (test 4.1%; control, 5.4, *p* = 0.048), while the other parameters were not significantly different (Table 2). The comparison of Δ values between baseline and 24 months after SPC in both groups showed that the test group experienced a significant reduction, among those, of BoP (*p* = 0.006), median PPD (*p* = 0.039), CAL (*p* = 0.042), and in sites with PPD ≥4 mm BoP+ (*p* = 0.005).

A mixed generalized linear regression analysis aimed at evaluating significant predictors of BoP at all follow‐up sessions (24‐month) from SPC baseline in both groups of patients found that reduced median BoP values over the follow‐up session using uni‐ and multi‐level estimation models were significantly influenced by the experimental treatment performed in the test group (*p* = 0.031, uni‐level; *p* = 0.033 multi‐level). For the model, positive coefficients identified a direct association between each covariate and the outcome variable, while negative coefficients indicated an inverse association. The uni‐level models showed that the reduced BoP was significantly influenced by smoking (*β* = 3.28; *p* = 0.019), high number of cigarettes/day (*β* = 4.81; *p* = 0.018), high median PPD (*β* = 4.28; *p* = 0.040), high no. of sites PPD 4‐5 mm (*β* = 2.29; *p* = 0.022), and by high no. of sites PPD > 6 mm (*β* = 2.87; *p* = 0.028). The multi‐level analysis showed that the reduced BoP was also significantly influenced by smoking (*β* = 3.61; *p* = 0.032), a high no. of cigarettes/day (*β* = 4.36; *p* = 0.045), a high median PPD (*β* = 3.37; *p* = 0.031), and a high no. of sites PPD 4–5 mm (*β* = 1.74; *p* = 0.029), no. of sites PPD 5–6 mm (*β* = 1.58; *p* = 0.036), and by high PPD > 6 mm (*β* = 2.74; *p* = 0.038) (Table 3). The VIF of the multi‐level model was 2.57, indicating moderate multicollinearity and the reliability of the coefficient estimates.

**TABLE 3:** Uni‐ and multi‐level mixed generalized linear regression model for BoP at all follow‐up sessions.

	Uni‐level model			Multi‐level model		
Parameter	*β*	95% CI	*p*‐value	*β*	95% CI	*p*‐value
**Fixed effects**						
*Treatment*	−3.31	−5.386; −2.713	0.031	−3.28	−2.459; −1.579	0.033
*Time*	1.35	0.087; 2.125	0.256	–	–	–
Treatment*Time	−3.84	−4.126; −3.845	0.214	–	–	–
Age	−2.59	−3.289; −1.431	0.292	–	–	–
Sex	0.96	−0.212; 2.336	0.068	–	–	–
Smoking	3.28	−3.214; 1.785	0.019	3.61	−2.141; 4.675	0.032
Cigarettes/day	4.81	2.984; 5.675	0.018	4.36	2.225; 4.581	0.045
Gingival biotype	−0.75	−1.415; 1.793	0.254	–	–	–
Patient compliance	3.39	1.498; 4.423	0.365	–	–	–
Median PPD	4.28	1.785; 5.459	0.040	3.37	1.445; 5.446	0.031
no. sites PPD < 4 mm	−4.31	−6.141; 2.786	0.541	–	–	–
no. sites PPD 4–5 mm	2.29	1.459; 2.789	0.022	1.74	0.228; 3.561	0.029
no. sites PPD 5–6 mm	3.12	0.596; 4.387	0.009	1.58	0.739; 3.118	0.036
no. sites PPD > 6 mm	2.87	1.863; 3.896	0.028	2.74	−3.429; 1.896	0.038
Median CAL	2.15	−1.658; 3.856	0.122	–	–	–
KT	−1.28	−2.558; −0.246	0.625	–	–	–
% FMPS	3.59	1.782; 4.563	0.334	–	–	–
**Random effects**						
Number of teeth per patient	0.765	0.484; 2.659	0.219	0.514	0.335; 2.356	0.259

*Note*: For treatment, control was set as a reference; for time, baseline was set as a reference; for sex, female was set as a reference; for gingival biotype, thick biotype was set as a reference; for patient compliance, regular SPC was set as a reference. Results were expressed as beta coefficient estimation (*β*), 95% confidence interval (95% CI), and *p*‐value.

Abbreviations: CAL, clinical attachment loss; GBI, gingival bleeding index; FMPS, full‐mouth plaque score; KT, keratinized tissue; PPD, probing pocket depth; SPC, supportive periodontal care.

The mixed generalized linear regression analysis evidenced also that the different number of teeth per each patient, inserted into the model such as random effect variable, did not influenced, in both uni‐ and multi‐level models, the reduction of median BoP at all follow‐up sessions (uni‐level, *β* = 0.765; *p* = 0.219; multi‐level, *β* = 0.514, *p* = 0.442).

## 4 DISCUSSION

Traditionally, SPC, consisting of effective tailored home hygiene and professional treatment, has served as an important phase of therapy for periodontitis patients and should be established after active stage therapy, for long‐term maintenance following active periodontal treatment.^13^ However, it has been reported that stable results from SPC are mainly influenced by tooth‐related factors, including BoP, PPD, tooth non‐vitality, and class II and III furcations, and the type of active periodontal treatment performed.^29^, ^30^ In the present study, the active stage of treatment was provided for all patients, using the same quadrant‐wise subgingival instrumentation approach. The choice was based on previous evidence that reported better outcomes of the traditional approach when compared with the one‐visit approach.^4^, ^5^, ^8^, ^29^ Significant reductions of the main periodontal outcomes assessed were achieved for all subjects.

The aim of the present study was to further analyze the long‐term results of active periodontal treatment obtained by comparing the efficacy of two SPC approaches in periodontitis patients on BoP reduction.

The choice of using BoP as the primary outcome variable was derived from previous evidence that reported that BoP represents an objective inflammatory parameter for the evaluation of SPC in the long‐term^15^, ^31^ in a prospective studies up to 53‐month follow‐up,^32^ and also by some other ones which reported how an increase of BoP is strictly correlated with a higher risk for disease progression.^33^, ^34^, ^35^

The results showed that, at the 24‐month follow‐up, both treatments were effective in reducing median periodontal outcomes, including BoP, PPD, CAL, and FMPS, with no significant tooth loss over time. However, the test treatment, which included subgingival debridement, was more effective than the control treatment in reducing median BoP, GBI, no. of pockets with PPD 4–5 mm, the number of pockets with PPD 5–6 mm, and with PPD ≥ 4 mm BoP+, % of pockets with PPD 5–6 mm, and % of pockets with PPD ≥6 mm. Moreover, the mixed linear regression model revealed that the treatment administered to the test group was more effective than the control in reducing the median BoP across all follow‐up sessions, even after considering several confounding variables.

It has been reported that SPC, which usually includes supragingival as well as subgingival instrumentation,^5^ together within a strict follow‐up program, maintains more favorable, stable results over time up to ≥7.5 years following active periodontal treatment.^36^ Some studies found that SPC accompanied by repeated subgingival instrumentation should be performed in order to prevent the regrowth of periodontal pathogens^37^ and reduce, in the short term, the need for further periodontal open flap surgery, especially when pockets ≥4 mm are present.^38^ However, the results of the present study conflict with a recent randomized clinical trial with 2‐year follow‐up, which reported no significant additional beneficial effects of subgingival debridement compared to the supragingival approach alone as an adjunct to SPC. A similar reduction in BoP of approximately 12.9% and a 14.2% reduction in bacterial proportions between the two techniques were reported after 2 years of follow‐up.^12^ In agreement, a recent Cochrane review that analyzed 307 periodontitis patients during SPC^39^ reported that there is insufficient evidence to determine the superiority of different protocols or adjunctive strategies to improve tooth maintenance during SPT. Perhaps the reorganization of the subgingival biofilm following active periodontal treatment, which impacts microbial succession in the subgingival ecosystem, may be less influenced by the SPT approach.^12^ At the same time, emerging evidence shows that the microbial succession seems to be influenced by several factors, including optimal oral hygiene regimens during SPC and associated with smoking cessation,^40^ that have been reported to significantly impact the different SPC approaches up to 3‐year follow‐up.^41^ Based on these findings, the present study was designed to further test the hypothesis that smoking could also influence the clinical outcomes of SPC in patients with periodontitis.

Results at 24 months of follow‐up, analyzed by the mixed linear regression model, showed that reduced BoP levels were significantly influenced by high median PPD, no. of sites PPD 4–5 mm, no. of sites PPD 5–6 mm, no. of sites PPD > 6 mm, % of sites PPD ≥4 mm, smoking, and by number of cigarettes per day. In this regard, a high number of cigarettes/day, together with greater numbers of deep pockets, negatively influenced the BoP reduction after treatment at all follow‐up sessions in both groups of patients. In agreement, an 11‐year follow‐up cross‐sectional study on residual pockets treatment showed a dose‐dependent effect of smoking on PPD (residual pockets increased substantially from 31.3% to 52.4% in heavy smokers) in comparison to non‐smoking patients.^33^ In this regard, a recent observational analysis showed that, despite a higher percentage of PI and PPD, BoP had a lower prevalence in smokers.^42^ Gugnani et al.,^43^ in a review of a total of 2743 studies, highlighted that heavy or current smoking was associated, in agreement with the results of the present study, with a significantly higher risk of worsening of periodontitis. This may be because smoking is capable of negatively influencing periodontitis and its treatment and subsequent SPC through different mechanisms^44^, ^45^ that reduce the overall host response in periodontal tissues.^46^, ^47^ Furthermore, it has been demonstrated that smoking is a powerful inducer of gingival inflammation in patients predisposed to periodontitis, and has an overall negative impact on periodontal health and related functions by accelerating tissue destruction.^20^

The present study was limited by a relatively small sample size. Also, a longer follow‐up would have been desirable to better evaluate the long‐term impact of SPC protocols and smoking in the analyzed sample. Moreover, during the different follow‐up sessions, both groups of patients may have been influenced by their different oral hygiene habits based on the treatment protocol to which they were assigned.

## 5 CONCLUSION

The present study demonstrated that an SPC protocol incorporating subgingival instrumentation yielded a statistically significant reduction in BoP and other periodontal outcomes compared to an SPC that excluded subgingival instrumentation. Smoking negatively influenced the reduced BoP at 24 months of follow‐up. Further studies with a larger sample will help to better evaluate the long‐term effectiveness of different SPC protocols in patients with periodontitis.

## AUTHOR CONTRIBUTIONS

Gaetano Isola conceived the research, planned and performed the experimental procedures, and wrote the manuscript. Gaetano Isola and Angela Angjelova performed the procedures. Angela Alibrandi performed the statistical analysis and concealed the data. Marco Annunziata and Gianluca Tartaglia validated the experimental results. Frank A. Scannapieco interpreted experimental results and wrote the manuscript. All the authors gave their final approval and agreed to be accountable for all aspects of the work.

## CONFLICT OF INTEREST STATEMENT

The authors declare that there are no conflicts of interest.

## Supporting information



Table S1: STROBE Guidelines.

Table S2: Periodontal characteristics of the analyzed sample at baseline, at each follow‐up session of the active periodontal treatment performed with quadrant‐wise subgingival instrumentation comparison between follow‐up sessions. Values are represented as mean such as median (CI 95%). PPD, probing pocket depth; CAL, clinical attachment loss; FMPS, full‐mouth plaque score. *, significance between baseline and 1‐month; ^†^, significance between baseline and 3‐months; ^‡^, significance between baseline and 6‐months; ^§^, significance between 1‐month and 3‐months; ^‖^, significance 1‐month and 6‐months; ^#^, significance between 3‐month and 6‐months. Significance set at *p* < 0.05.

## Footnotes

* G POWER, Universität Düsseldorf, Düsseldorf, Germany

† UNC‐15, Hu‐Friedy, Milan, Italy

‡ Meridol, CP‐GABA, Hamburg, Germany

§ Tepe, Malmo, Sweden

‖ Satelec Ultrasonics, Acteon, Varese, Italy

¶ Instrument PS, EMS Electro Medical System S.A., Pero, Italy

# SPSS 22.0, IBM, Bologna, Italy
