# Supplementary material for: Effectiveness of two supportive periodontal care protocols and outcome predictors during periodontitis: A randomized controlled trial
Source: J Periodontol. 2025 Nov 7;97(3):435–49. doi: 10.1002/jper.70007 (PMC13111776; doi:10.1002/jper.70007)
Supplement: Supplementary file 2 — Table S2: Periodontal characteristics of the analyzed sample at baseline, at each follow‐up session of the active periodontal treatment performed with quadrant‐wise subgingival instrumentation comparison between follow‐up sessions. Values are represented as mean such as median (CI 95%). PPD, probing pocket depth; CAL, clinical attachment loss; FMPS, full‐mouth plaque score. *, significance between baseline and 1‐month; †, significance between baseline and 3‐months; ‡, significance between baseline and 6‐months; §, significance between 1‐month and 3‐months; ‖, significance 1‐month and 6‐months; #, significance between 3‐month and 6‐months. Significance set at p < 0.05. [file JPER-97-435-s002.docx]

Supplementary Table 2: Periodontal characteristics of the analysed sample at baseline and at each follow-up session of the active periodontal treatment performed with quadrant-wise subgingival instrumentation comparison between follow-up sessions. Values are represented such as median (IQR). PPD, Probing Pocket Depth; CAL, Clinical Attachment Loss; FMBS, Full mouth bleeding score; FMPS, Full mouth plaque score. Bonferroni corrections between time points; *, significance between follow-up sessions with a significant p-value at adjusted alpha level of 0.008. P<0.05 among all follow-up sessions.

| **Variable** | **Treated (n= 58)** |
| --- | --- |
| **Mean PPD (mm)** |  |
| Baseline | 4.79 (4.2-4.9) |
| 1 month | 3.59 (3.2-3.8) * |
| 3 months | 2.85 (2.5-2.9) |
| 6 months - retreatment | 3.35 (2.7-3.5) * |
| P-value intragroup | 0.006 |
| **No. pockets PD ≥4 mm BoP+** |  |
| Baseline | 77.5 (69.5-85.6) |
| 1 month | 36.5 (21.3-44.5) * |
| 3 months | 12.2 (8.6-19.3) * |
| 6 months - retreatment | 14.5 (10.5-19.5) * |
| P-value intragroup | 0.025 |
| **No. pockets PPD ≥4 mm** |  |
| Baseline | 58.8 (41.1-61.4) |
| 1 month | 35.6 (25.6-49.3) * |
| 3 months | 27.5 (18.5-36.3) * |
| 6 months - retreatment | 17.5 (9.6-19.5) * |
| P-value intragroup | 0.027 |
| **No. pockets PPD ≥5 mm** |  |
| Baseline | 38.5 (26.5-47.2) |
| 1 month | 30.3 (20.3-41.2) * |
| 3 months | 24.4 (18.5-38.4) * |
| 6 months - retreatment | 18.9 (8.5-21.5) * |
| P-value intragroup | 0.044 |
| **No. pockets PPD ≥6 mm** |  |
| Baseline | 13.5 (7.9-16.6) |
| 1 month | 6.9 (4.1-8.4) * |
| 3 months | 3.5 (2.9-5.2) * |
| 6 months - retreatment | 2.9 (1.9-3.6) * |
| P-value intragroup | <0.001 |
| **% PD <4 mm** |  |
| Baseline | 41.6 (30.2-48.5) |
| 1 month | 49.5 (40.6-52.3) * |
| 3 months | 62.5 (41.2-74.6) * |
| 6 months - retreatment | 78.4 (62.5-89.6) * |
| P-value intragroup | 0.048 |
| **% PD ≥4 mm** |  |
| Baseline | 59.6 (42.3-69.5) |
| 1 month | 35.6 (28.5-41.3) * |
| 3 months | 24.4 (17.5-29.6) * |
| 6 months - retreatment | 22.3 (14.5-26.5) * |
| P-value intragroup | 0.045 |
| **% PD ≥5 mm** |  |
| Baseline | 37.8 (29.5-44.5) |
| 1 month | 26.1 (20.6-35.4) * |
| 3 months | 14.2 (9.1-17.5) * |
| 6 months - retreatment | 10.5 (6.9-13.5) * |
| P-value intragroup | 0.029 |
| **% PD ≥6 mm** |  |
| Baseline | 11.8 (7.5-14.5) |
| 1 month | 7.7 (5.4-8.9) * |
| 3 months | 4.1 (2.5-7.1) * |
| 6 months - retreatment | 3.5 (2.9-4.1) * |
| P-value intragroup | 0.051 |
| **Mean CAL (mm)** |  |
| Baseline | 4.85 (3.1-5.6) |
| 1 month | 3.84 (3-4.2) * |
| 3 months | 3.32 (2.7-3.6) * |
| 6 months - retreatment | 3.38 (2.8-3.5) * |
| P-value intragroup | 0.021 |
| **No. pockets CAL ≥4 mm** |  |
| Baseline | 61.3 (52.4-65.6) |
| 1 month | 37.5 (24.3-51.2) * |
| 3 months | 31.2 (21.2-38.4) * |
| 6 months - retreatment | 15.6 (11.6-18.4) * |
| P-value intragroup | 0.029 |
| **No. pockets CAL ≥5 mm** |  |
| Baseline | 83.5 (72.4-91.3) |
| 1 month | 58.2 (42.6-67.4) * |
| 3 months | 33.1 (24.5-39.4) * |
| 6 months - retreatment | 19.1 (13.5-27.4) * |
| P-value intragroup | 0.017 |
| **No. pockets CAL ≥6 mm** |  |
| Baseline | 13.1 (7.9-17.5) |
| 1 month | 6.5 (4.1-8.2) * |
| 3 months | 3.5 (2.1-4.2) * |
| 6 months - retreatment | 3.1 (2.1-4.2) * |
| P-value intragroup | 0.012 |
| **% CAL <4 mm** |  |
| Baseline | 53.6 (41.2-59.6) |
| 1 month | 58.5 (42.5-61.2) * |
| 3 months | 67.5 (54.1-75.6) * |
| 6 months - retreatment | 79.4 (61.2-89.6) * |
| P-value intragroup | 0.023 |
| **% CAL ≥4 mm** |  |
| Baseline | 57.5 (45.2-62.3) |
| 1 month | 46.5 (35.2-54.2) * |
| 3 months | 33.4 (20.2-41.3) * |
| 6 months - retreatment | 20.5 (17.5-26.2) * |
| P-value intragroup | 0.012 |
| **% CAL ≥5 mm** |  |
| Baseline | 56.1 (41.2-62.3) |
| 1 month | 40.1 (31.2-52.3) * |
| 3 months | 30.4 (21.2-40.2) * |
| 6 months - retreatment | 19.5 (14.5-26.3) * |
| P-value intragroup | 0.011 |
| **% CAL ≥6 mm** |  |
| Baseline | 10.5 (7.1-12.3) |
| 1 month | 9.4 (7.5-10.2) * |
| 3 months | 8.1 (6.5-10.4) * |
| 6 months - retreatment | 3.4 (2.1-4.2) * |
| P-value intragroup | 0.025 |
| **FMBS (%)** |  |
| Baseline | 49.5 (32.5-52.3) |
| 1 month | 29.5 (20.2-36.5) * |
| 3 months | 38.6 (15.6-49.5) * |
| 6 months - retreatment | 32.6 (20.5-38.2) * |
| P-value intragroup | 0.041 |
| **FMPS (%)** |  |
| Baseline | 51.4 (41.2-60.2) |
| 1 month | 27.5 (19.5-36.5) * |
| 3 months | 23.5 (14.2-28.5) * |
| 6 months - retreatment | 18.9 (12.5-36.5) * |
| P-value intragroup | 0.017 |
